# Supplementary material for: The Immune Landscape of Colorectal Cancer
Source: Cancers (Basel). 2021 Nov 4;13(21):5545. doi: 10.3390/cancers13215545 (PMC8583221; doi:10.3390/cancers13215545)
Supplement: Supplementary file 1 [file cancers-13-05545-s001.zip › Figure S1.pdf]

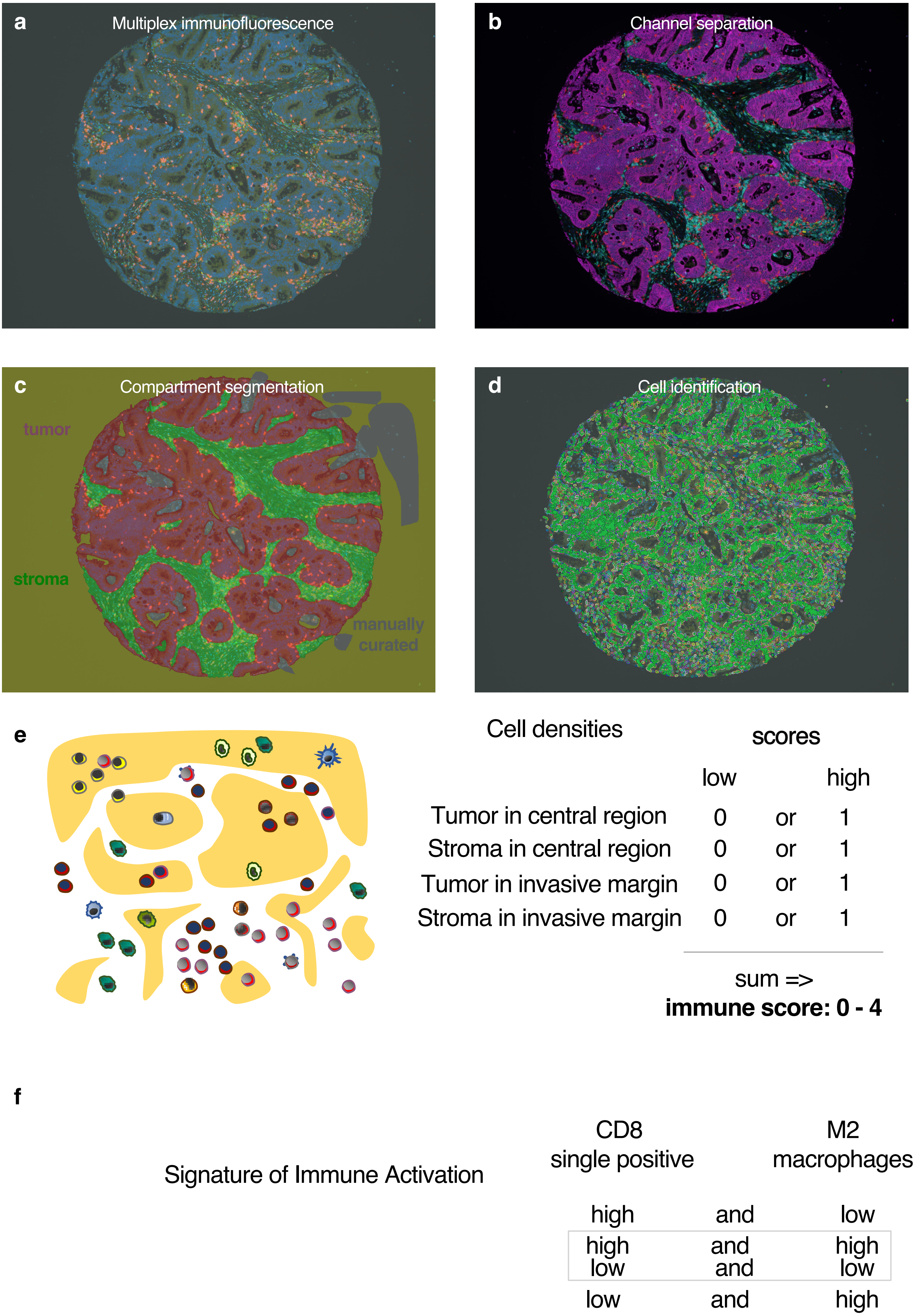
a
Multiplex immunofluorescence
b
Channel separation
c
Compartment segmentation
tumor
stroma
manually curated
d
Cell identification
e
Cell densities
scores
low
high
Tumor in central region 0 or 1
Stroma in central region 0 or 1
Tumor in invasive margin 0 or 1
Stroma in invasive margin 0 or 1
sum =>
immune score: 0 - 4
f
Signature of Immune Activation
CD8 single positive
M2 macrophages
high and low
high and high
low and low
low and high
