# Supplementary material for: The Immune Landscape of Colorectal Cancer
Source: Cancers (Basel). 2021 Nov 4;13(21):5545. doi: 10.3390/cancers13215545 (PMC8583221; doi:10.3390/cancers13215545)
Supplement: Supplementary file 1 [file cancers-13-05545-s001.zip › Figure S4.pdf]

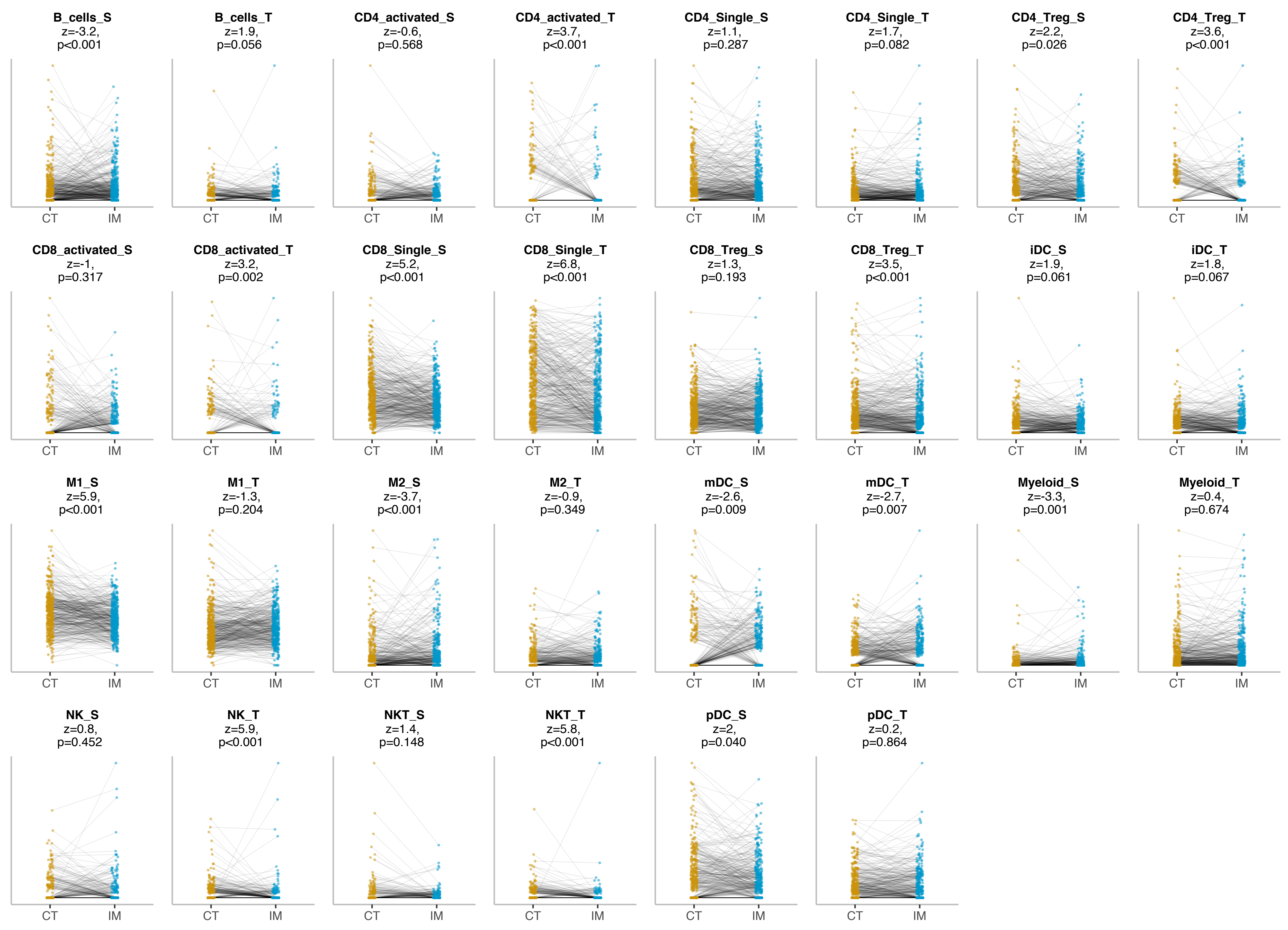

B_cells_S
z=-3.2,
p<0.001
B_cells_T
z=1.9,
p=0.056
CD4_activated_S
z=-0.6,
p=0.568
CD4_activated_T
z=3.7,
p<0.001
CD4_Single_S
z=1.1,
p=0.287
CD4_Single_T
z=1.7,
p=0.082
CD4_Treg_S
z=2.2,
p=0.026
CD4_Treg_T
z=3.6,
p<0.001
CD8_activated_S
z=-1,
p=0.317
CD8_activated_T
z=3.2,
p=0.002
CD8_Single_S
z=5.2,
p<0.001
CD8_Single_T
z=6.8,
p<0.001
CD8_Treg_S
z=1.3,
p=0.193
CD8_Treg_T
z=3.5,
p<0.001
iDC_S
z=1.9,
p=0.061
iDC_T
z=1.8,
p=0.067
M1_S
z=5.9,
p<0.001
M1_T
z=-1.3,
p=0.204
M2_S
z=-3.7,
p<0.001
M2_T
z=-0.9,
p=0.349
mDC_S
z=-2.6,
p=0.009
mDC_T
z=-2.7,
p=0.007
Myeloid_S
z=-3.3,
p=0.001
Myeloid_T
z=0.4,
p=0.674
NK_S
z=0.8,
p=0.452
NK_T
z=5.9,
p<0.001
NKT_S
z=1.4,
p=0.148
NKT_T
z=5.8,
p<0.001
pDC_S
z=2,
p=0.040
pDC_T
z=0.2,
p=0.864
CT
IM
