# Supplementary material for: The Immune Landscape of Colorectal Cancer
Source: Cancers (Basel). 2021 Nov 4;13(21):5545. doi: 10.3390/cancers13215545 (PMC8583221; doi:10.3390/cancers13215545)
Supplement: Supplementary file 1 [file cancers-13-05545-s001.zip › Table S1.pdf]

Table S1. Baseline clinicopathological characteristics. Values are the number (percentage) unless indicated otherwise. Percentages may not add to 100% due to rounding.

**Characteristics**	All panels (N = 536)	TIL panel (N = 520)	NK/MF panel (N = 508)	APC Panel (N = 498)
Age				
Average age in years ± SD	69.69 ± 12.01	69.82 ± 12.02	69.77 ± 11.90	69.79 ± 11.82
≤ 75 years old	351 (65.5)	338 (65)	331 (65.2)	327 (65.7)
> 75 years old	185 (34.5)	182 (35)	177 (34.8)	171 (34.3)
Gender				
Male	290 (54.1)	282 (54.2)	274 (53.9)	273 (54.8)
Female	246 (45.9)	238 (45.8)	234 (46.1)	225 (45.2)
Localization				
Right Colon	185 (34.5)	183 (35.2)	177 (34.8)	169 (33.9)
Left Colon	184 (34.3)	182 (35)	178 (35)	165 (33.1)
Rectum	167 (31.2)	155 (29.8)	153 (30.1)	164 (32.9)
T Stage				
0	7 (1.3)	3 (0.6)	5 (1)	7 (1.4)
1	39 (7.3)	39 (7.5)	37 (7.3)	35 (7)
2	66 (12.3)	61 (11.7)	60 (11.8)	65 (13.1)
3	314 (58.6)	309 (59.4)	304 (59.8)	291 (58.4)
4	101 (18.8)	99 (19)	95 (18.7)	92 (18.5)
Missing data	9 (1.7)	9 (1.7)	7 (1.4)	8 (1.6)
N Stage				
0	283 (52.8)	273 (52.5)	266 (52.4)	261 (52.4)
1+	243 (45.3)	237 (45.6)	236 (46.5)	228 (45.8)
Missing data	10 (1.9)	10 (1.9)	6 (1.2)	9 (1.8)
M Stage				
0	450 (84)	437 (84)	426 (83.9)	421 (84.5)
1	80 (14.9)	77 (14.8)	77 (15.2)	71 (14.3)
Missing data	6 (1.1)	6 (1.2)	5 (1)	6 (1.2)
Differentiation Grade				
Low	374 (69.8)	368 (70.8)	356 (70.1)	353 (70.9)
High	87 (16.2)	84 (16.2)	83 (16.3)	79 (15.9)
Missing data	75 (14)	68 (13.1)	69 (13.6)	66 (13.3)
Neural Invasion				
No	375 (70)	361 (69.4)	352 (69.3)	347 (69.7)
Yes	80 (14.9)	80 (15.4)	78 (15.4)	73 (14.7)
Missing data	81 (15.1)	79 (15.2)	78 (15.4)	78 (15.7)
Vascular Invasion				
No	344 (64.2)	331 (63.7)	326 (64.2)	320 (64.3)
Yes	133 (24.8)	132 (25.4)	125 (24.6)	120 (24.1)
Missing data	59 (11)	57 (11.0)	57 (11.2)	58 (11.6)
MSS T Status				
MSS	454 (84.7)	441 (84.8)	432 (85)	423 (84.9)
MSI-H	68 (12.7)	66 (12.7)	64 (12.6)	63 (12.7)
Missing data	14(2.6)	13 (2.5)	12 (2.4)	12 (2.4)
BRAF Mutation				
No	210 (39.2)	206 (39.6)	201 (39.6)	194 (39)
Yes	46 (8.6)	46 (8.8)	45 (8.9)	42 (8.4)

Missing data	280 (52.2)	268 (51.5)	262 (51.5)	262 (52.6)
MSI: BRAF Mutation				
MSI-H + BRAF Mutation	21 (30.9)	21 (31.8)	21 (32.8)	20 (31.7)
MSI-H + BRAF WT	29 (42.6)	17 (25.8)	17 (26.6)	17 (27)
Missing data	18 (26.5)	28 (42.4)	26 (40.6)	26 (41.3)
Neoadjuvant treatment in colon cancer				
No	364 (98.6)	360 (98.6)	350 (98.6)	329 (98.5)
Yes	2 (0.5)	2 (0.5)	2 (0.6)	2 (0.6)
Missing data	3 (0.8)	3 (0.8)	3 (0.8)	3 (0.9)
Neoadjuvant treatment in rectal cancer				
No	52 (31.1)	51 (32.9)	48 (31.4)	50 (30.5)
Yes	102 (61.1)	91 (58.7)	93 (60.8)	101 (61.6)
Missing data	13 (7.8)	13 (8.4)	12 (7.8)	13 (7.9)
Neoadjuvant Treatment Type (Rectum only)				
No treatment	54 (32.3)	52 (33.5)	49 (32.1)	52 (31.7)
scRT+delayed surgery	33 (19.8)	32 (20.6)	31 (20.3)	32 (19.5)
scRT+immediate surgery	30 (18)	29 (18.7)	28 (18.3)	30 (18.3)
scRT+CT	15 (9.0)	10 (6.5)	11 (7.2)	15 (9.1)
CRT	10 (6.0)	8 (5.2)	10 (6.5)	10 (6.1)
CT	1 (0.6)	1 (0.6)	1 (0.7)	1 (0.6)
Long-course RT	1 (0.6)	1 (0.6)	1 (0.7)	1 (0.6)
Missing data	23 (13.8)	22 (14.2)	22 (14.4)	23 (14)
