# Supplementary material for: The Immune Landscape of Colorectal Cancer
Source: Cancers (Basel). 2021 Nov 4;13(21):5545. doi: 10.3390/cancers13215545 (PMC8583221; doi:10.3390/cancers13215545)
Supplement: Supplementary file 1 [file cancers-13-05545-s001.zip › Table S2.pdf]

Table S2. Immune scores in tumors with different clinical and pathological characteristics. Chi-square test was used for statistical analysis.

Clinicopathological characteristics		N (cases)	TIL panel: CD4 single						CD4+CD45RO+						CD4 Treg					
	Immune cell score		0	1	2	3	4	p value	0	1	2	3	4	p value	0	1	2	3	4	p value
Tumor location	Right colon	427	106	34	19	10	14	0,025*	121	33	20	6	3	0,329	97	32	33	13	8	0,014*
	Left colon		123	17	19	10	11		135	17	17	4	7		123	19	19	11	8	
	Rectum		52	7	2	0	3		48	8	6	1	1		48	4	9	0	3	
	Colon		229	51	38	20	25	0,038*	256	50	37	10	10	0,931	220	51	52	24	16	0,072
	Rectum		52	7	2	0	3		48	8	6	1	1		48	4	9	0	3	
	Right Colon		106	34	19	10	14	0,036*	121	33	20	6	3	0,101	97	32	33	13	8	0,006**
	Left Colon + Rectum		175	24	21	10	14		183	25	23	5	8		171	23	28	11	11	
pT stage	T0	419	1	0	0	0	0	0,823	0	1	0	0	0	0,308	1	0	0	0	0	0,620
	T1		22	5	2	2	3		24	2	7	0	1		23	2	6	0	3	
	T2		25	2	4	2	1		24	4	4	1	1		24	4	3	1	2	
	T3		159	39	29	11	21		177	41	24	9	8		148	39	43	18	11	
	T4		66	12	5	5	3		72	10	7	1	1		64	10	9	5	3	
pN stage	N0	419	136	27	18	11	18	0,560	144	28	25	6	7	0,597	124	30	33	9	14	0,099
	N1		139	29	22	9	10		153	30	17	5	4		138	25	26	15	5	
pM stage	M0	422	231	48	32	17	25	0,723	248	49	37	9	10	0,712	218	47	53	20	15	0,804
	M1		47	9	8	3	2		53	9	4	2	1		46	8	7	4	4	
Differentiation Grade	High	367	40	12	9	3	9	0,163	47	13	5	4	4	0,108	36	10	15	6	6	0,094
	Low		198	38	28	14	16		213	35	33	6	7		191	39	38	13	13	
Vasc. Invasion	No	378	168	35	27	15	20	0,609	182	36	33	7	7	0,271	162	32	40	17	14	0,756
	Yes		81	13	9	4	6		86	17	6	2	2		74	15	15	6	3	
Neur. Invasion	No	367	195	40	30	15	23	0,786	213	41	36	7	6	0,306	179	41	48	19	16	0,122
	Yes		46	8	5	2	3		49	10	2	1	2		49	5	6	3	1	
MSI status	MSI	416	37	14	2	3	7	0,030*	41	8	7	4	3	0,191	29	12	12	6	4	0,041*
	MSS		239	41	37	16	20		257	47	35	7	7		233	40	49	16	15	
BRAF	WT	233	122	21	19	12	14	0,054	134	27	18	3	6	0,571	121	20	23	14	10	0,261
	Mut.		30	11	2	0	2		32	6	5	2	0		27	4	10	4	0	
MSI: BRAF status	WT	38	8	5	1	1	2	0,576	10	4	2	0	1	0,385	6	6	1	3	1	0,103
	Mut.		13	6	0	0	2		14	2	3	2	0		11	3	6	1	0	
Age	≤ 75	427	174	35	33	13	16	0,122	188	35	36	7	5	0,045*	170	34	37	18	12	0,800
	> 75		107	23	7	7	12		116	23	7	4	6		98	21	24	6	7	

Clinicopathological characteristics		N (cases)	TIL panel: CD8 single						CD8+CD45RO+						CD8 Treg						B cells					
	Immune cell score		0	1	2	3	4	p value	0	1	2	3	4	p value	0	1	2	3	4	p value	0	1	2	3	4	p value
Tumor location	Right colon	427	68	40	34	27	14	0,038*	103	36	28	8	8	0,283	79	31	33	15	25	0,030*	115	25	31	9	3	0,139
	Left colon		65	31	56	12	16		117	35	20	5	3		101	35	23	6	15		121	31	17	3	8	
	Rectum		22	9	23	5	5		46	7	7	1	3		38	14	4	2	6		46	8	8	1	1	
	Colon		133	71	90	39	30	0,409	220	71	48	13	11	0,322	180	66	56	21	40	0,237	236	56	48	12	11	0,794
	Rectum		22	9	23	5	5		46	7	7	1	3		38	14	4	2	6		46	8	8	1	1	
	Right Colon		68	40	34	27	14	0,004**	103	36	28	8	8	0,200	79	31	33	15	25	0,004**	115	25	31	9	3	0,046*
	Left Colon + Rectum		87	40	79	17	21		163	42	27	6	6		139	49	27	8	21		167	39	25	4	9	
pT stage	T0	419	0	0	0	1	0	0,430	0	0	1	0	0	0,241	0	1	0	0	0	0,457	0	1	0	0	0	0,001**
	T1		13	6	11	1	3		17	7	7	1	2		17	3	7	0	7		17	3	7	1	6	
	T2		10	6	10	5	3		22	10	1	0	1		15	8	5	1	5		21	5	5	1	2	
	T3		88	50	68	30	23		160	45	34	12	8		131	48	36	18	26		175	41	32	8	3	
	T4		40	18	21	7	5		61	16	11	1	2		48	20	11	4	8		61	14	12	3	1	
pN stage	N0	419	64	42	52	31	21	0,009**	121	41	30	11	7	0,115	96	33	38	14	29	0,011*	132	37	29	4	8	0,210
	N1		87	37	59	13	13		141	36	24	3	5		116	46	22	9	16		144	26	26	9	4	
pM stage	M0	422	117	70	98	38	30	0,051	213	71	47	10	12	0,111	173	66	55	20	39	0,217	233	56	42	11	11	0,492
	M1		36	8	14	6	5		50	6	7	4	2		42	13	4	3	7		45	8	13	2	1	
Differentiation Grade	High	367	27	16	12	10	8	0,250	41	12	11	4	5	0,288	34	11	6	7	15	0,026*	44	9	15	4	1	0,241
	Low		105	53	84	31	21		186	53	38	10	7		148	57	46	15	28		196	44	38	7	9	
Vasc. Invasion	No	378	89	46	74	32	24	0,115	161	48	38	8	10	0,836	126	53	42	16	28	0,666	175	41	32	7	10	0,402
	Yes		50	24	24	10	5		74	21	12	3	3		61	21	14	4	13		72	17	18	5	1	
Neur. Invasion	No	367	103	55	81	38	26	0,064	185	56	43	8	11	0,544	150	56	46	18	33	0,609	200	45	40	9	9	0,796
	Yes		32	12	15	3	2		46	10	6	1	1		33	16	8	2	5		43	7	10	3	1	
MSI status	MSI	416	14	15	6	18	10	<0,001***	23	13	17	5	5	<0,001***	11	10	14	11	17	<0,001***	36	10	11	3	3	0,421
	MSS		138	63	102	25	25		241	60	37	8	7		202	69	44	12	26		239	53	43	9	9	
BRAF	WT	233	66	36	56	19	11	0,253	122	34	26	2	4	0,215	94	41	30	5	18	0,080	121	31	24	7	5	0,576
	Mut.		19	13	7	3	3		25	8	7	1	4		20	8	5	5	7		27	5	9	3	1	
MSI: BRAF status	WT	38	2	3	0	10	2	0,016*	5	4	7	0	1	0,519	1	5	4	1	6	0,206	7	4	3	1	2	0,917
	Mut.		9	5	2	2	3		8	5	4	1	3		6	3	4	4	4		10	4	5	1	1	
Age	≤ 75	427	93	45	82	30	21	0,124	171	52	33	7	8	0,728	145	49	36	15	26	0,673	173	42	38	8	10	0,524
	> 75		62	35	31	14	14		95	26	22	7	6		73	31	24	8	20		109	22	18	5	2	

Clinicopathological characteristics		NK/MF panel																														
		N (cases)	NK cells						NKT cells						M1						M2						Myeloid					
	Immune cell score		0	1	2	3	4	*p value*	0	1	2	3	4	*p value*	0	1	2	3	4	*p value*	0	1	2	3	4	*p value*	0	1	2	3	4	*p value*
Tumor location	Right colon	413	123	30	16	3	5	0,900	127	31	14	2	3	0,775	45	35	53	16	28	0,207	110	18	26	11	12	**0,029***	107	37	22	6	5	**0,009****
	Left colon		125	30	17	2	2		141	24	8	1	2		62	35	48	9	22		129	19	19	9	0		123	24	27	2	0	
	Rectum		46	6	6	1	1		46	8	5	0	1		15	9	22	8	6		44	5	6	4	1		50	5	4	0	1	
	Colon		248	60	33	5	7	0,747	268	55	22	3	5	0,902	107	70	101	25	50	0,249	239	37	45	20	12	0,851	230	61	49	8	5	0,074
	Rectum		46	6	6	1	1		46	8	5	0	1		15	9	22	8	6		44	5	6	4	1		50	5	4	0	1	
	Right Colon		123	30	16	3	5	0,782	127	31	14	2	3	0,479	45	35	53	16	28	0,487	110	18	26	11	12	**0,003****	107	37	22	6	5	**0,006****
	Left Colon + Rectum		171	36	23	3	3		187	32	13	1	3		77	44	70	17	28		173	24	25	13	1		173	29	31	2	1	
pT stage	T0	407	1	0	0	0	0	0,875	1	0	0	0	0	0,448	0	0	0	0	1	0,106	1	0	0	0	0	0,967	1	0	0	0	0	0,830
	T1		19	8	4	0	1		26	3	1	0	2		10	1	10	2	9		24	1	5	2	0		21	2	8	1	0	
	T2		21	4	6	1	0		22	6	3	0	1		11	4	12	2	3		22	5	3	1	1		22	5	5	0	0	
	T3		187	37	22	4	5		193	41	19	1	1		76	52	70	22	35		173	26	30	18	8		170	44	33	5	3	
	T4		60	17	7	1	2		67	13	3	2	2		22	22	29	7	7		58	10	13	3	3		61	15	7	2	2	
pN stage	N0	408	140	33	21	4	4	0,855	148	32	16	0	6	0,023	60	35	59	17	31	0,720	140	18	26	11	7	0,933	134	32	31	3	2	0,574
	N1		151	31	18	2	4		164	29	10	3	0		61	44	62	15	24		139	23	25	13	6		142	33	22	5	4	
pM stage	M0	409	245	55	30	4	6	0,567	255	59	19	2	5	0,142	98	67	101	28	46	0,945	234	38	41	17	10	0,326	229	60	42	5	4	0,099
	M1		46	10	9	2	2		57	4	6	1	1		23	12	19	5	10		46	4	9	7	3		49	5	10	3	2	
Differentiation Grade	High	355	51	14	6	0	1	0,566	53	12	6	0	1	0,949	13	15	24	5	15	0,135	41	7	12	7	5	0,099	48	14	8	1	1	0,925
	Low		200	41	29	6	7		215	44	18	1	5		87	54	84	24	34		197	28	36	16	6		189	45	39	6	4	
Vasc. Invasion	No	365	179	38	30	3	8	**0,040***	194	38	19	1	6	0,125	73	49	80	26	30	0,416	179	25	30	19	5	0,210	175	44	33	4	2	0,375
	Yes		77	22	5	3	0		80	21	4	2	0		35	25	27	6	14		71	11	13	5	7		72	17	11	4	3	
Neur. Invasion	No	355	206	45	31	5	6	0,443	215	50	20	2	6	0,640	88	52	92	27	34	0,145	201	29	34	22	7	0,197	196	52	37	5	3	0,336
	Yes		42	14	3	1	2		49	9	3	1	0		20	18	11	5	8		41	8	8	1	4		44	6	8	3	1	
MSI status	MSI	402	50	9	3	0	0	0,28	41	15	6	0	0	0,129	12	8	19	3	20	**<0,001*****	33	5	12	6	6	**0,001****	37	18	5	2	0	**0,026***
	MSS		238	53	35	6	8		263	47	21	3	6		107	70	100	29	34		245	35	37	17	6		236	47	45	6	6	
BRAF	WT	226	124	29	22	3	4	0,844	139	27	11	2	3	0,824	58	41	45	14	24	0,439	124	19	23	11	5	0,392	118	33	23	4	4	0,335
	Mut.		30	9	3	1	1		30	8	4	1	1		8	11	15	3	7		26	5	6	3	4		29	9	3	3	0	
MSI: BRAF status	WT	38	13	3	1	0	0	0,894	13	4	0	0	0	0,257	3	3	2	1	8	0,303	10	1	3	2	1	0,941	11	4	1	1	0	0,948
	Mut.		16	3	2	0	0		13	5	3	0	0		4	4	8	1	4		11	1	4	2	3		12	4	1	1	0	
Age	≤ 75	413	188	37	27	5	6	0,458	206	37	15	1	4	0,532	86	48	74	22	33	0,397	194	22	30	12	5	**0,024***	184	44	29	3	3	0,244
	> 75		106	29	12	1	2		108	26	12	2	2		36	31	49	11	23		89	20	21	12	8		96	22	24	5	3	

Clinicopathological characteristics		APC Panel																		
		N (cases)	iDC						mDC						pDC					
	Immune cell score		0	1	2	3	4	*p value*	0	1	2	3	4	*p value*	0	1	2	3	4	*p value*
Tumor location	Right colon	395	87	28	29	12	13	0,135	83	44	28	10	4	0,086	107	27	13	8	14	**0,022***
	Left colon		90	36	23	9	5		84	37	26	7	9		94	24	21	13	11	
	Rectum		43	6	10	2	2		21	17	17	7	1		26	9	14	4	10	
	Colon		177	64	52	21	18	0,171	167	81	54	17	13	**0,031***	201	51	34	21	25	**0,008****
	Rectum		43	6	10	2	2		21	17	17	7	1		26	9	14	4	10	
	Right Colon		87	28	29	12	13	0,169	83	44	28	10	4	0,770	107	27	13	8	14	0,091
	Left Colon + Rectum		133	42	33	11	7		105	54	43	14	10		120	33	35	17	21	
pT stage	T0	388	1	0	0	0	0	0,582	0	1	0	0	0	0,229	1	0	0	0	0	0,116
	T1		20	4	4	0	2		18	2	8	1	1		16	2	3	1	8	
	T2		24	3	4	2	0		11	12	8	2	0		18	4	8	2	1	
	T3		133	44	35	16	12		110	62	44	14	10		136	40	30	16	18	
	T4		37	19	17	5	6		45	21	8	7	3		52	14	6	6	6	
pN stage	N0	388	110	32	28	11	8	0,920	81	55	36	10	7	0,229	105	26	26	11	21	0,315
	N1		107	37	32	12	11		105	42	31	14	7		121	33	21	12	12	
pM stage	M0	390	181	60	48	20	18	0,811	147	86	60	23	11	0,086	180	57	43	20	27	0,055
	M1		36	10	12	3	2		39	11	9	1	3		45	3	5	4	6	
Differentiation Grade	High	341	31	16	13	2	5	0,270	38	15	7	4	3	0,382	44	10	5	5	3	0,461
	Low		156	49	38	18	13		123	73	51	17	10		155	42	35	17	25	
Vasc. Invasion	No	346	138	46	37	14	11	0,704	115	64	49	10	8	0,007	135	42	33	13	23	**0,048***
	Yes		48	21	17	8	6		48	25	9	13	5		69	12	7	8	4	
Neur. Invasion	No	336	155	55	41	16	12	0,249	130	70	50	18	11	0,722	161	45	32	15	26	0,102
	Yes		24	11	12	5	5		28	17	6	4	2		36	8	7	6	0	
MSI status	MSI	385	34	8	7	5	6	0,158	25	16	13	3	3	0,821	36	8	3	4	9	0,142
	MSS		180	61	54	18	12		156	80	57	21	11		184	51	45	21	24	
BRAF	WT	216	91	31	27	13	13	0,162	87	44	24	14	6	0,375	111	24	19	12	9	0,446
	Mut.		14	14	7	3	3		23	7	9	1	6		21	9	5	2	4	
MSI: BRAF status	WT	37	13	0	0	0	4	**0,008****	6	7	3	1	0	0,356	11	3	0	1	2	0,819
	Mut.		8	5	4	2	1		12	3	3	1	1		12	2	1	2	3	
Age	≤ 75	395	143	41	40	10	17	0,062	113	70	47	16	5	0,072	139	41	36	11	24	0,080
	> 75		77	29	22	13	3		75	28	24	8	9		88	19	12	14	11	
