# Supplementary material for: The Immune Landscape of Colorectal Cancer
Source: Cancers (Basel). 2021 Nov 4;13(21):5545. doi: 10.3390/cancers13215545 (PMC8583221; doi:10.3390/cancers13215545)
Supplement: Supplementary file 1 [file cancers-13-05545-s001.zip › Table S3.pdf]

Table S3. Distribution of clinicopathological parameters in tumors within 'inflamed' and 'Immune desert' clusters. See also Figure 2c.

**Characteristics**	Immune dessert (N = 228)	Inflamed (N = 145)	p-value
Age			
≤ 75 years old	144 (63.2)	92 (64.4)	0.955
> 75 years old	84 (36.8)	53 (36.6)	
Gender			
Male	123 (53.9)	77 (53.1)	0.873
Female	105 (46.1)	68 (46.9)	
Localization			
Right Colon	80 (35.1)	80 (55.2)	0.001
Left Colon	106 (46.5)	49 (33.8)	
Rectum	42 (18.4)	16 (11.0)	
T Stage			
0-1	15 (6.8)	14 (9.7)	0.437
2	19 (8.6)	12 (8.3)	
3	136 (61.3)	94 (64.8)	
4	52 (23.4)	25 (17.5)	
Missing data	6	0	
N Stage			
0	101 (44.7)	78 (54.9)	0.056
1	125 (55.3)	64 (45.1)	
Missing data	2	3	
M Stage			
0	188 (82.8)	122 (85.9)	0.430
1	39 (17.2)	20 (14.1)	
Missing data	1	3	
Differentation Grade			
Low	165 (86.8)	96 (71.6)	0.001
High	25 (13.2)	38 (28.4)	
Missing data	38	11	
Neural Invasion			
No	148 (75.5)	113 (89.7)	0.005
Yes	43 (22.5)	13 (10.3)	
Missing data	37	19	
Vascular Invasion			
No	131 (67.2)	100 (76.3)	0.074
Yes	64 (32.8)	31 (23.7)	
Missing data	33	14	
MSS Status			
MSS	207 (92.4)	102 (72.9)	<0.001
MSI-H	17 (7.6)	38 (27.1)	
Missing data	4	5	
BRAF Mutation			
No	102 (82.3)	62 (77.5)	0.403
Yes	22 (17.7)	18 (22.5)	
Missing data	104	65	
