# Supplementary material for: The Immune Landscape of Colorectal Cancer
Source: Cancers (Basel). 2021 Nov 4;13(21):5545. doi: 10.3390/cancers13215545 (PMC8583221; doi:10.3390/cancers13215545)
Supplement: Supplementary file 1 [file cancers-13-05545-s001.zip › Table S4.pdf]

Table S4. Differences in immune cell distribution across cancer samples from patients with different clinicopathological characteristics. The direction of the association is illustrated by the location of the asterisks.

*- p<0.05; **-p<0.01; ***-p<0.001.

			Location		pT stage		pN stage		pM stage		Differentiation Grade		Vasc. invasion		Neur. invasion		MSI status		BRAF		MSI: BRAF status		Age	
			Colon	Rectum	T0-T3	T4	N0	N1	M0	M1	High	Low	No	Yes	No	Yes	MSI	MSS	WT	Mutation	WT	Mutation	≤ 75	> 75
TIL panel N (cases)	CT	S	348	50	303	89	195	197	330	64	70	273	250	108	285	62	60	327	180	45	16	21	254	144
		T	348	50	303	89	195	197	330	64	70	273	250	108	285	62	60	327	180	45	16	21	254	144
	IM	S	317	36	274	79	169	182	297	54	65	245	222	103	259	56	59	285	163	42	17	19	226	127
		T	317	36	274	79	169	182	297	54	65	245	222	103	259	56	59	285	163	42	17	19	226	127
CD4 single	CT	S																						
		T																						
	IM	S	**				*				*													
		T	*										*											
CD4+CD45RO+	CT	S																						
		T			*				**															
	IM	S			*												*							
		T									*						**							
CD4_Treg	CT	S																						
		T													*		**							
	IM	S			*		**										*							
		T									**						**							
CD8 single	CT	S																						
		T							*				*		*		**							
	IM	S			*		*				**		*		*		***							
		T			**		***		*				**		***		**							
CD8+CD45RO+	CT	S					**																	
		T															**							
	IM	S					*										***							*
		T					*				**						***			*				
CD8_Treg	CT	S					*										**							
		T			**		***		*				*		*		***			*				
	IM	S					**										***							*
		T			*		***								*		***							**
B cells	CT	S																						
		T					*																	
	IM	S			**												**							
		T									*						***			*				

			Location		pT stage		pN stage		pM stage		Differentiation Grade		Vasc. invasion		Neur. invasion		MSI status		BRAF		MSI: BRAF status		Age	
			Colon	Rectum	T0-T3	T4	N0	N1	M0	M1	High	Low	No	Yes	No	Yes	MSI	MSS	WT	Mutation	WT	Mutation	≤ 75	> 75
NK/MF panel N (cases)		S	331	48	289	85	186	189	310	65	70	255	239	100	272	58	58	310	171	43	16	21	242	137
		T	331	48	289	85	186	189	310	65	70	255	239	100	272	58	58	310	171	43	16	21	242	137
	IM	S	302	34	260	76	158	176	283	51	63	232	213	96	248	51	55	271	155	40	16	18	215	121
		T	302	34	260	76	158	176	283	51	63	232	213	96	248	51	55	271	155	40	16	18	215	121
NK cells	CT	S																**						
		T																						
	IM	S					**																	
		T					**																	
NKT cells	CT	S		**																				
		T		*																				
	IM	S					*																	
		T					*								*									
M1	CT	S																						
		T									***						***			*				
	IM	S																						
		T									**						***							
M2	CT	S		*																				
		T								*	*						*							*
	IM	S									***			*			**			*				***
		T	*								**						*							**
Myeloid	CT	S						*																
		T																						*
	IM	S									**			*			**							**
		T																						*

			Location		pT stage		pN stage		pM stage		Differentiation Grade		Vasc. invasion		Neur. invasion		MSI status		BRAF		MSI: BRAF status		Age	
			Colon	Rectum	T0-T3	T4	N0	N1	M0	M1	High	Low	No	Yes	No	Yes	MSI	MSS	WT	Mutation	WT	Mutation	≤ 75	> 75
APC panel N (cases)	CT	S	317	50	281	81	175	187	305	58	62	256	233	94	263	55	53	305	170	38	17	129	238	129
		T	317	50	281	81	175	187	305	58	62	256	233	94	263	55	53	305	170	38	17	129	238	129
	IM	S	284	37	248	73	147	172	271	48	56	227	204	89	235	49	51	261	152	36	14	118	203	118
		T	284	37	248	73	147	172	271	48	56	227	204	89	235	49	51	261	152	36	14	118	203	118
iDC	CT	S																						
		T														*								
	IM	S								*				**		*		**						
		T									*									**				
mDC	CT	S																						
		T																						
	IM	S			**							*							*					
		T																						
pDC	CT	S		***			*						*											
		T		**									*											
	IM	S		*									**										*	
		T																					**	
