# Supplementary material for: The Immune Landscape of Colorectal Cancer
Source: Cancers (Basel). 2021 Nov 4;13(21):5545. doi: 10.3390/cancers13215545 (PMC8583221; doi:10.3390/cancers13215545)
Supplement: Supplementary file 1 [file cancers-13-05545-s001.zip › Table S5.pdf]

Table S5. Univariable associations of immune scores with OS in a complete cohort of therapy-naïve patients. See also Figure 3a.

Immune score	HR (95% CI)	P value	Q value
CD4_Single	0.83 (0.62-1.1)	0.189	0.405
CD4_CD45RO	0.78 (0.58-1.1)	0.107	0.268
CD4_Treg	0.87 (0.67-1.2)	0.338	0.563
CD8_Single	0.64 (0.49-0.84)	0.001	0.014
CD8_CD45RO	0.84 (0.64-1.1)	0.225	0.422
CD8_Treg	0.78 (0.58-1)	0.101	0.268
B_cells	1 (0.78-1.4)	0.818	0.876
NK	1.1 (0.8-1.4)	0.669	0.836
NKT	1.1 (0.8-1.5)	0.576	0.785
M1	0.99 (0.76-1.3)	0.946	0.946
M2	1.5 (1.2-2)	0.001	0.014
Myeloid	1.1 (0.83-1.5)	0.514	0.771
iDC	1.3 (0.99-1.7)	0.057	0.268
mDC	0.8 (0.61-1)	0.101	0.268
pDC	0.96 (0.7-1.3)	0.796	0.876
