# Supplementary material for: The Immune Landscape of Colorectal Cancer
Source: Cancers (Basel). 2021 Nov 4;13(21):5545. doi: 10.3390/cancers13215545 (PMC8583221; doi:10.3390/cancers13215545)
Supplement: Supplementary file 1 [file cancers-13-05545-s001.zip › Table S6.pdf]

Table S6. Association of immune scores with RFS in stage I-III colon cancer, univariable Cox regression models. See also Figure 3b, left panel.

Immune score	HR (95% CI)	P value	Q value
CD4_Single	1.1 (0.77-1.5)	0.659	0.760
CD4_CD45RO	0.88 (0.61-1.3)	0.472	0.708
CD4_Treg	0.87 (0.62-1.2)	0.412	0.687
CD8_Single	0.68 (0.49-0.94)	0.021	0.158
CD8_CD45RO	0.92 (0.66-1.3)	0.604	0.760
CD8_Treg	0.8 (0.57-1.1)	0.217	0.651
B_cells	0.95 (0.67-1.3)	0.762	0.777
NK	0.92 (0.64-1.3)	0.657	0.760
NKT	1.2 (0.81-1.7)	0.398	0.687
M1	1 (0.76-1.5)	0.777	0.777
M2	1.7 (1.3-2.4)	<0.001	0.016
Myeloid	1.2 (0.84-1.6)	0.357	0.687
iDC	1.4 (0.97-1.9)	0.070	0.349
mDC	0.83 (0.59-1.2)	0.281	0.687
pDC	0.76 (0.5-1.1)	0.192	0.651
