# Supplementary material for: The Immune Landscape of Colorectal Cancer
Source: Cancers (Basel). 2021 Nov 4;13(21):5545. doi: 10.3390/cancers13215545 (PMC8583221; doi:10.3390/cancers13215545)
Supplement: Supplementary file 1 [file cancers-13-05545-s001.zip › Table S7.pdf]

Table S7. Association of immune scores with RFS in stage I-III colon cancer, multivariable Cox regression model, adjusted to clinicopathological parameters. See also Figure 3b, right panel.

Immune score	HR (95% CI)	P value
CD4_Single	1.14 (0.69-1.89)	0.616
CD4_CD45RO	1.13 (0.65-1.95)	0.666
CD4_Treg	1.02 (0.58-1.82)	0.934
CD8_Single	0.64 (0.41-0.98)	0.039
CD8_CD45RO	1.07 (0.67-1.69)	0.788
CD8_Treg	0.83 (0.5-1.36)	0.455
B_cells	1.46 (0.97-2.2)	0.073
NK	1.07 (0.63-1.81)	0.813
NKT	1.31 (0.78-2.2)	0.303
M1	1.04 (0.68-1.58)	0.854
M2	1.43 (0.86-2.39)	0.166
Myeloid	0.92 (0.55-1.55)	0.752
iDC	1.21 (0.84-1.75)	0.31
mDC	0.92 (0.63-1.34)	0.654
pDC	1 (0.64-1.57)	0.998
T stage 1-2 (ref)		
T stage 3	1.4 (0.69-2.84)	0.354
T stage 4	2.56 (1.16-5.63)	0.02
N stage 0 (ref)		
N stage 1	2.89 (1.82-4.59)	<0.001
Colon side (left vs right)	1.09 (0.72-1.64)	0.69
Differentiation grade high (ref)		
Differentiation grade low	0.8 (0.48-1.34)	0.403
Differentiation grade missing	0.74 (0.36-1.53)	0.417
Age at diagnosis (>75 years old)	2.09 (1.36-3.22)	0.001
Elective or Acute surgery: Acute (ref)		
Elective or Acute surgery: Elective	0.54 (0.35-0.85)	0.007
Adjuvant treatment: missing (ref)		
Adjuvant treatment: NO	0.35 (0.22-0.55)	<0.001
Adjuvant treatment: YES	0.25 (0.13-0.45)	<0.001
