# Supplementary material for: The Immune Landscape of Colorectal Cancer
Source: Cancers (Basel). 2021 Nov 4;13(21):5545. doi: 10.3390/cancers13215545 (PMC8583221; doi:10.3390/cancers13215545)
Supplement: Supplementary file 1 [file cancers-13-05545-s001.zip › Table S8.pdf]

Table S8. Univariable associations of immune scores with OS in stage VI therapy-naïve colon cancer patients. See also Figure 3c.

Immune score	HR (95% CI)	P value	Q value
CD4_Single	0.38 (0.2-0.7)	0.002	0.031
CD4_CD45RO	0.79 (0.43-1.5)	0.462	0.77
CD4_Treg	0.62 (0.35-1.1)	0.099	0.297
CD8_Single	0.55 (0.32-0.95)	0.033	0.185
CD8_CD45RO	0.86 (0.49-1.5)	0.615	0.828
CD8_Treg	0.89 (0.47-1.7)	0.718	0.828
B_cells	0.75 (0.44-1.3)	0.287	0.538
NK	0.59 (0.33-1)	0.064	0.241
NKT	0.62 (0.31-1.3)	0.19	0.407
M1	1.5 (0.89-2.5)	0.129	0.323
M2	1.2 (0.68-2)	0.589	0.828
Myeloid	1.1 (0.65-2)	0.662	0.828
iDC	0.98 (0.58-1.7)	0.943	0.943
mDC	0.56 (0.32-0.97)	0.037	0.185
pDC	1.1 (0.59-2)	0.819	0.878
