# Supplementary figures and images for: The Immune Landscape of Colorectal Cancer
Source: Cancers (Basel). 2021 Nov 4;13(21):5545. doi: 10.3390/cancers13215545 (PMC8583221; doi:10.3390/cancers13215545)

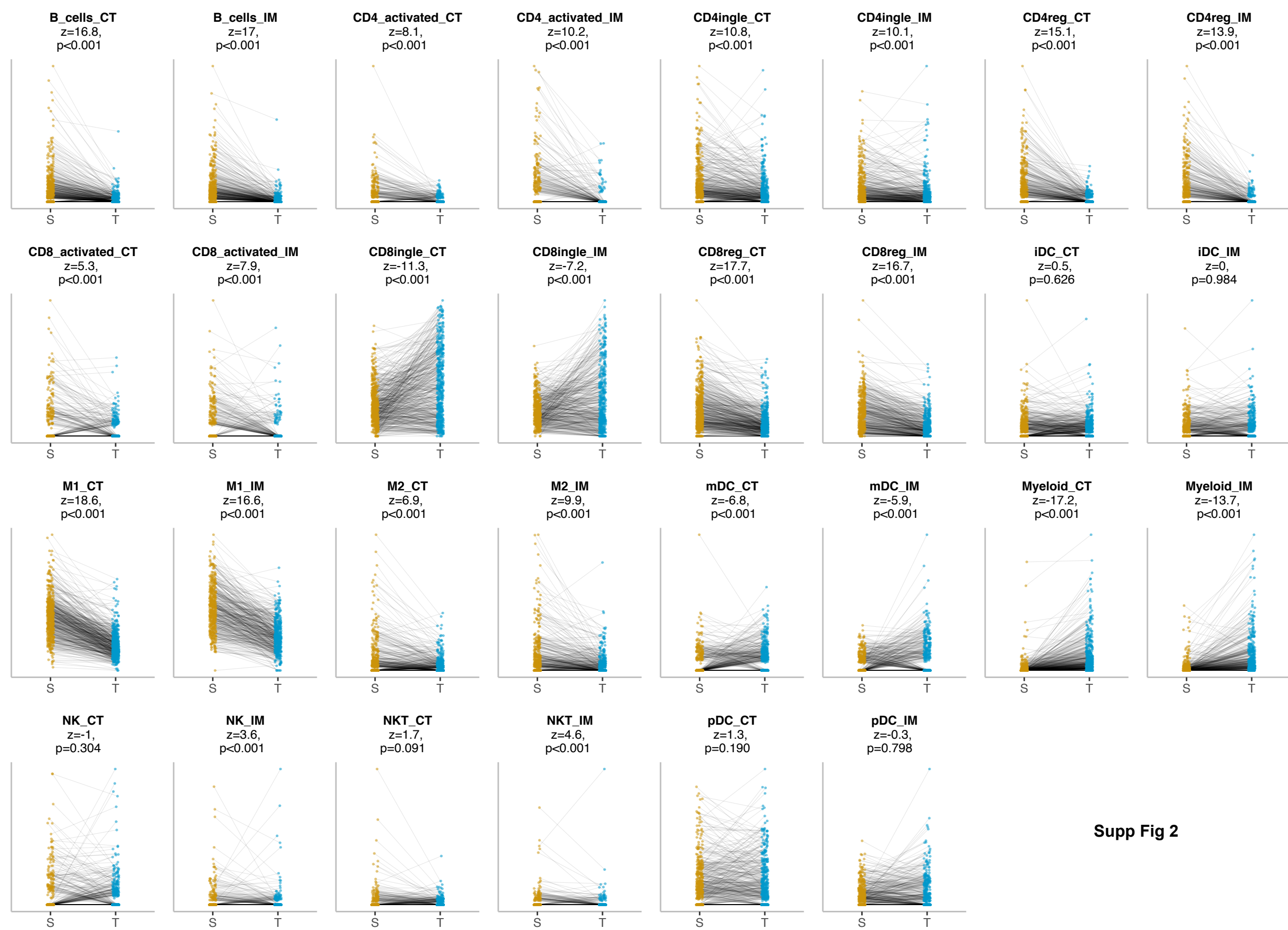

Supplement: Supplementary file 1 [file cancers-13-05545-s001.zip › Figure S2.pdf]

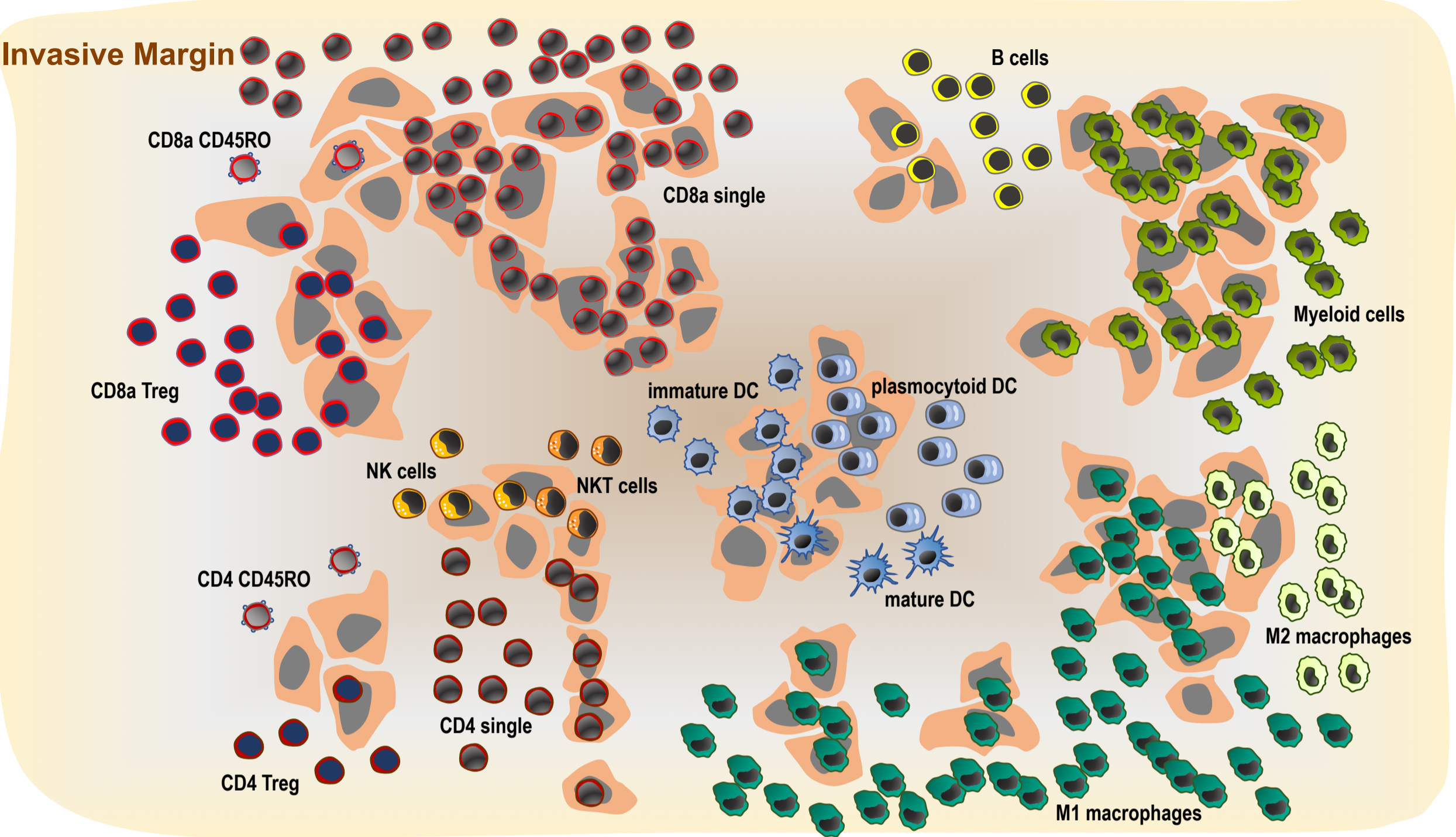

Supp Fig 3

Supplement: Supplementary file 1 [file cancers-13-05545-s001.zip › Figure S3.pdf]

Figure S5

a

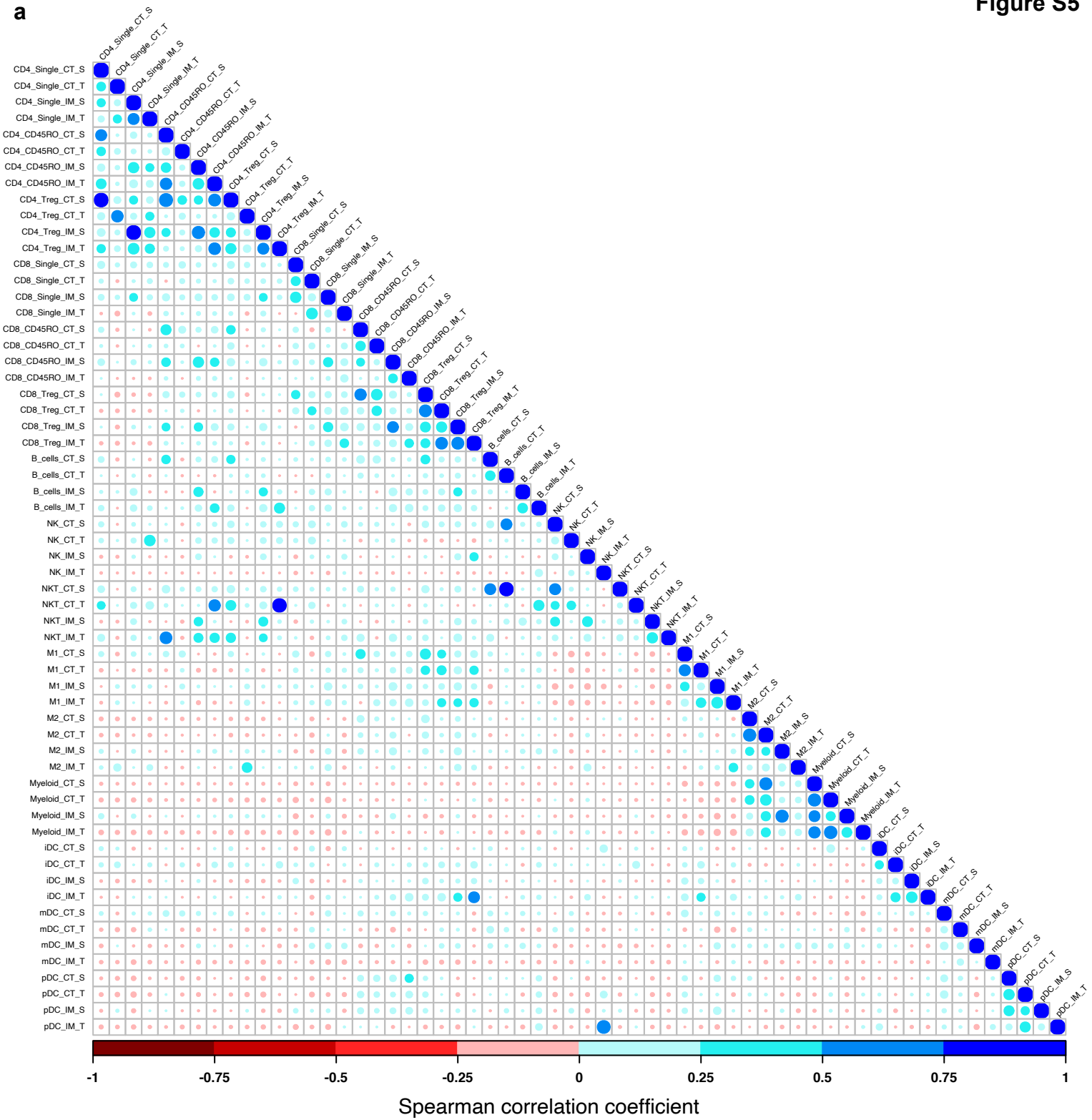

b

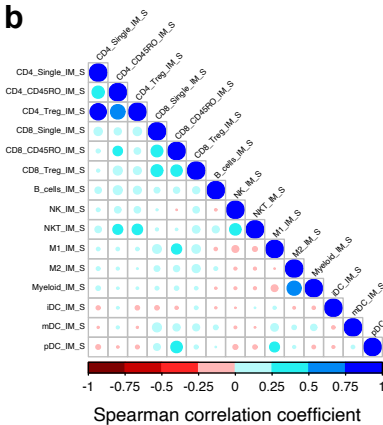

c

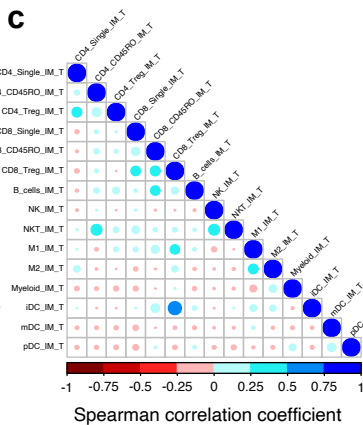

d

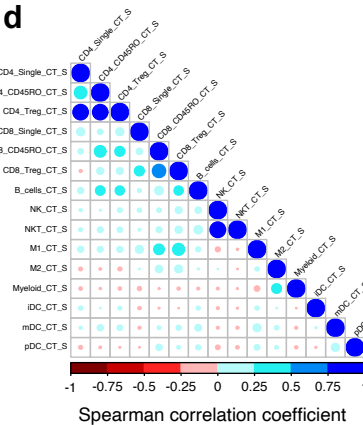

e

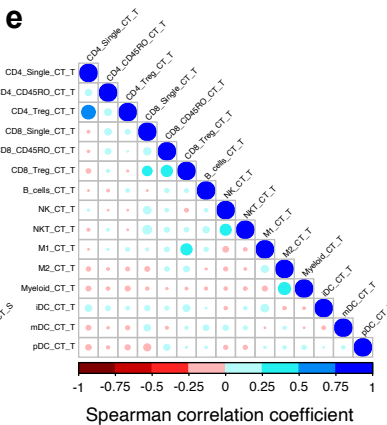

Supplement: Supplementary file 1 [file cancers-13-05545-s001.zip › Figure S5.pdf]
